# Dr. Magendie on the Organs of Absorption

**Published:** 1811-01

**Authors:** Jesse Foot

**Affiliations:** Dean-street, Soho


### *To the Editors of the Medical and Physical Journal.*

Gentlemen,

I Request you will insert, in your widely circulated Journal, the following statement. It is meant merely to put in my claim to a *discovery*, without the least offence intended to Mr. Ramsden.

Yours, &c.
JESSE FOOT.

*Dean-street, Soho, Dec.* 15, 1810.

ON perusing the Table of Contents in Mr. Ramsden's late Publication, my attention was particularly arrested by the following article. "Description of a Membranous Fence at "the Aperture of the Urethra *hitherto* UNNOTICED; a common cause of derangement of the Urethra." And on coming to page 36 of the work, which is referred to by the Table of Contents, the explanation of the discovery, so important in Mr. Ramsden's opinion, commences with the following paragraph:—

"There is, however, another cause which is very commonly productive of that derangement of the urinary passage now under consideration, and which I believe has not been noticed, viz. an unnatural diminution of the external opening of the urethra.

"In the majority of persons who apply for assistance under complaints of the testicle or of the urethra, which are not to be traced to gonorrhœal inflammation, the urethra will be found to be more contracted at its orifice than at any other part of the canal. This state of its extremity is very different from the stricture which is sometimes situated immediately within the aperture, as well as from that appearance of this part, which is called the blind urethra.

"The diminution of the orifice of the urethra is occasioned by a membranous fence, which partially closes up the lips of the extremity of the canal; it may sometimes be an original mal-formation: yet I apprehend it is for the most part produced by cohesion during infancy, and is analogous to the union we sometimes see between the prepuce and glans penis of the male child, and between the nymphæ in the female.

"Unimportant as this diminution of the extremity of the urethra may at first appear, many valuable facts may be deduced from it. It is the only instance, I believe, in which an immediate cause of spasmodic stricture in that canal can be be demonstrated, by occasioning too strong and too frequent action of the adjacent muscles upon its diameter: at the same time it affords an excellent illustration of the manner in which other causes, although more remote, may either predispose the urethra to stricture, or establish in its membrane a state of latent irritation."

Mr. Ramsden proceeds to state the consequences of this hitherto unnoticed cause of derangement of the urethra, along a page or two further; but as my object is merely to establish a prior claim to this hitherto unnoticed cause of derangement of the urethra, I shall content myself with proving my priority of right.

In the year 1803, a new edition of a work entitled, CASES OF A SUCCESSFUL PRACTICE OF VESICÆ LOTURA FOR THE CURE OF DISEASED BLADDER, was published, with a second Part, containing more CASES; and also Cases of diseased Affections from Phymosis, and on too SMALL A PERFORATION OT THE GLANS PENIS.

In the second part of this Edition, page 10, are the following remarks.

"Amongst the causes, as the cases offer, I shall demonstrate to a physical certainty, that natural phymosis, and too small a perforation of the glans penis, are causes of dangerous affections of urethra, bladder, and kidnies, and that nothing but the operation fitting for either will be the foundation of a cure. This has not been generally attended to, or what has been generally done has been so done as to be disgraceful to surgery. Still I must be so candid as to allow that I know there are some, and these among my friends, who are apprised of the importance of this subject, and who treat it as I have done."

And in page 84 of the same work, is the following Case and Observations thereon.

"CASE XVIII.

"*Of too small a Perforation of the Glans Penis.*

"A gentleman of the law, aged twenty-two, who had resided in Jamaica two years, was forced to return from thence, for a complaint in the kidnies and bladder, accompanied with a difficulty in evacuating his urine.

"He told me, that he had enjoyed perfect health till within these two years; but that ever since he was in a warm climate, these affections were rapidly advancing, till they had arrived to that excess in which they were now experienced. To all appearance, no one could be, with any chance of recovery, in a worse state. Upon attempting to pass pass a bougie, I found there was, in limine, a great difficulty in passing it, I could scarcely get the smallest bougie to enter the perforation or orifice of the glans. There also appeared a scar, left from a former ulcer, not directly upon the orifice of the glans, but on one side of it. Upon attempting again and again to pass up a bougie of that size, which any natural orifice would have readily admitted, I still found it impracticable.

"I suggested to him, that the smallness of the orifice might be the cause of all his symptoms; that it was my firm opinion it was the cause; and that dilating, or enlarging the orifice, was the sine qua non of his ever getting well. The orifice was dilated after the following manner.

"I passed the largest bougie I could into the urethra, and made my incision, upon the orifice enlarging it on its lower end. I considered that enough was done, when I could pass a large bougie. The point of a bougie was passed up twice a day till it healed. After this, I left his situation, for some time, to its natural action, without attempting any thing more, to see how far the relief thus given would be productive. The whole of the symptoms gradually abated. He discharged at length his urine in a fair stream, at proper distances, and in proper quantities. But notwithstanding this effect of the operation, it is very singular to relate, that I was never able to get a bougie into the bladder, though nothing was omitted for at least six months, that I thought would conduce to it. The orifice was dilated two years ago, and he has ever since continued free from any return of symptoms.

"OBSERVATION.

"I have relieved two more cases of this sort. They were taken in time, so that the symptoms produced in consequence of them, had not gone such a length as they had in the above case. But it was the symptoms they had in consequence of the smallness of the perforation, and not the smallness of the perforation, that induced these two patients to apply to me."

Since the above Publication in 1803, I have witnessed some of the most distressing Cases which ever fell under my care, produced from natural Phymosis, and from the *Smallness of the Perforation of the Glans Penis.*

For the present I thus most humbly put in my claim of priority. But I mean at my leisure to make observations upon Mr. Ramsden's performance, when I shal' give the Cases above alluded to at some length, and supported by the most positive evidence.

I trust Mr. Ramsden will have the fairness voluntarily to restore to me this feather, as in critical justice, every bird should have his proper one.

G 2 I will

I will no further prosecute the subject, for the present, but give Mr. Ramsden time for recollection, and conclude with the following observation from Dr. Johnson.

"Even a man whose genius qualifies him for great undertakings, must be content to learn, at least, from books, the present state of human knowledge, that he may not ascribe to himself the invention of arts generally known; weary his attention with experiments of which the event has been long registered; and waste, in attempts which have already succeeded or miscarried, that time which might have been spent with usefulness and honor upon new undertakings."

